# Interleukin-1 Inhibition and Fatigue in Primary Sjögren's Syndrome – A Double Blind, Randomised Clinical Trial

**DOI:** 10.1371/journal.pone.0030123

**Published:** 2012-01-10

**Authors:** Katrine Brække Norheim, Erna Harboe, Lasse G. Gøransson, Roald Omdal

**Affiliations:** 1 Clinical Immunology Unit, Department of Internal Medicine, Stavanger University Hospital, Stavanger, Norway; 2 Institute of Internal Medicine, University of Bergen, Bergen, Norway; University of Perugia, Italy

## Abstract

**Objectives:**

Fatigue is a major cause of disability in primary Sjögren's syndrome (pSS). Fatigue has similarities with *sickness behaviour* in animals; the latter mediated by pro-inflammatory cytokines, in particular interleukin (IL)-1, acting on neuronal brain cells. We hypothesised that IL-1 inhibition might improve fatigue in pSS patients; thus, we examined the effects and safety of an IL-1 receptor antagonist (anakinra) on fatigue.

**Methods:**

Twenty-six pSS patients participated in a double-blind, placebo-controlled parallel group study. Patients were randomised to receive either anakinra or a placebo for four weeks. Fatigue was evaluated by a fatigue visual analogue scale and the Fatigue Severity Scale. The primary outcome measure was a group-wise comparison of the fatigue scores at week 4, adjusted for baseline values. Secondary outcome measures included evaluation of laboratory results and safety. The proportion of patients in each group who experienced a 50% reduction in fatigue was regarded as a post-hoc outcome. All outcomes were measured at week 4.

**Results:**

There was no significant difference between the groups in fatigue scores at week 4 compared to baseline after treatment with anakinra. However, six out of 12 patients on anakinra versus one out of 13 patients on the placebo reported a 50% reduction in fatigue VAS (p = 0.03). There were two serious adverse events in each group.

**Conclusions:**

This randomised, double-blind, placebo-controlled trial of IL-1 blockade did not find a significant reduction in fatigue in pSS in its primary endpoint. A 50% reduction in fatigue was analysed post-hoc, and significantly more patients on the active drug than on placebo reached this endpoint. Although not supported by the primary endpoint, this may indicate that IL-1 inhibition influences fatigue in patients with pSS.

**Trial registration:**

ClinicalTrials.gov NCT00683345

## Introduction

Primary Sjögren's syndrome (pSS) is a chronic autoimmune disease with an estimated prevalence of 0.05%–0.5% [1], [2]. Clinical characteristics are dryness of the mouth and eyes – xerostomia and keratoconjunctivitis sicca. Histopathological examination reveals lymphocytic infiltration in exocrine glands, sometimes with ectopic germinal centre formation [3]. The majority of patients have autoantibodies against the ribonuclein particles SSA/Ro and/or SSB/La. Patients frequently have extraglandular manifestations such as muscle and joint pain, neuropathy, and fatigue [4]. Fatigue is a major cause of disability [5]. Recently it was reported that 85% of pSS patients experience fatigue, and 40% of the patients report fatigue as their most severe symptom [6]. It is well known that mood disorders influence fatigue, but in pSS fatigue occurs in non-depressed as well as depressed individuals [7], [8]. Other factors influencing fatigue in pSS are pain, sleep disorders, learned helplessness, and possibly neuroendocrine disturbances and autonomic dysfunction [9].

In search of biological mechanisms for fatigue we and others have found *sickness behaviour* in animals to be a relevant model [10]. In animals, this behaviour is an adaptive and appropriate response to infection and inflammation, and is characterised by increased sleep, decreased activity, social withdrawal, and loss of appetite [10]. A number of animal studies have demonstrated that sickness behaviour is signalled through interleukin (IL)-1 receptors on neurons in the brain [11]. This is exemplified by intraperitoneal (IP) or intracerebroventricular (ICV) injections of IL-1β or lipopolysaccharide (LPS), which leads to sickness behaviour within a few hours [12]. There is no such effect following LPS-injections in IL-1 knockout mice [13]. IL-1 exists in a membrane bound form (IL-1α) and a circulating form (IL-1β) and has two receptors: IL-1RI induces signal transduction, while IL-1RII functions as a decoy receptor [14]. A naturally occurring IL-1 receptor antagonist (IL-1Ra) inhibits IL-1 signalling by competitive binding to IL-1RI [15]. IL-1Ra crosses the blood-brain barrier (BBB) and recombinant IL-1Ra administered systemically may inhibit the effect of IL-1 in the brain [16]. Injection of recombinant IL-1Ra in animals before injection of LPS diminishes sickness behaviour [17].

Injection of IL-1 in humans leads to fever, fatigue, and nausea [18], [19]. We recently demonstrated that increased activation in the IL-1 system, as detected by raised levels of IL-1Ra in cerebrospinal fluid (CSF), is associated with more fatigue in pSS [7].

The mapping of biological pathways associated with fatigue is important in order to understand the phenomenon and to point out possible new treatment targets. Anakinra is a recombinant IL-1Ra used in the treatment of rheumatoid arthritis (RA), adult Still's disease, and autoinflammatory diseases. It is administered daily by a subcutaneous injection. Administration of anakinra reduced fatigue in a non-blinded pilot trial in RA patients [20]. We hypothesised that fatigue in pSS is mediated through activation of IL-1 receptors in the brain analogue to sickness behaviour in animals. Inhibition of these receptors may lead to a reduction in fatigue; thus, the objective of the current study was to investigate the effect of IL-1 inhibition on fatigue in pSS.

## Materials and Methods

The protocol for this trial and supporting CONSORT checklist are available as supporting information; see Checklist S1 and Protocol S1.

### Ethics statement

All patients gave written informed consent to participate, and the study (ClinicalTrials.gov number NCT00683345) was approved by the regional ethics committee, REK-Nord, Norway, and carried out in compliance with the principles expressed in the Declaration of Helsinki.

### Patients

We reviewed the medical records of all pSS patients between the ages of 18–80 years who lived in Rogaland County, Norway in 2008. Eligible participants were of Western European descent, met the 2002 American-European Consensus Group criteria for pSS [21], and spoke Norwegian. Exclusion criteria were: untreated comorbid conditions influencing fatigue (i.e. untreated hypothyroidism, heart failure), significant depression (score ≥20 on the Beck Depression Inventory (BDI) [22]), no fatigue measured by the Fatigue Severity Scale (FSS) [23] (score ≤3), anaemia (haemoglobin <100 g/L), neutropenia (neutrophil count <1.5×10^9^/L), actual or recurrent infections, pregnancy or lactation, or concurrent treatment with biological drugs.

**Figure 1:**
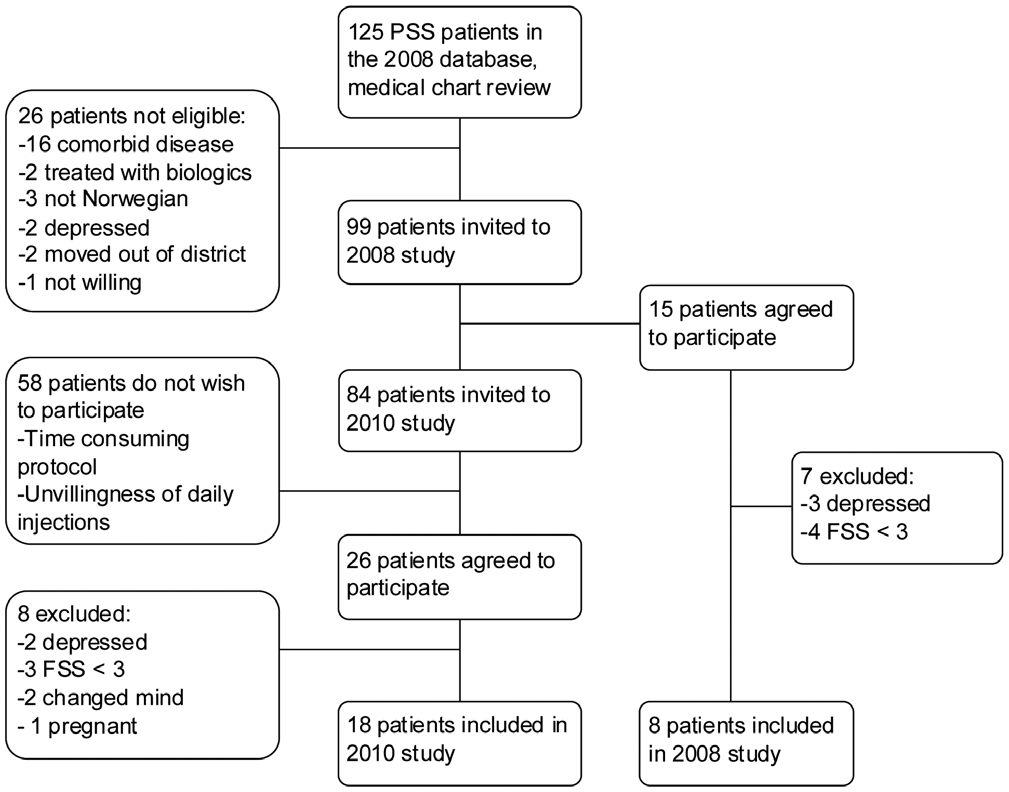
Flowchart of inclusion in the study. The pilot-study was conducted in 2008 and the main study in 2010. All patients underwent the same procedures. PSS, primary Sjögren's syndrome; FSS, Fatigue Severity Scale.

One hundred and twenty-five pSS patients were registered in Rogaland County in 2008, of which 26 were deemed to be ineligible. Thus, 99 patients were invited by letter to participate in the study. For logistic reasons, the study was conducted in two phases, a pilot study in 2008 and the main study in 2010. Of the 15 patients who agreed to participate in 2008, eight were included. The remaining 84 patients were invited to participate in the 2010 study and 18 were included. A flow chart visualizing the inclusion and reasons for non-inclusion is provided in Figure 1. In total, 26 of 125 (21%) pSS patients, 19 women (73%) and seven men (27%), were randomised to receive treatment.

### Study design and visits

The study was designed as a single centre, prospective, randomised, double-blind, placebo-controlled parallel group trial. Patients had a total of five study visits; baseline (inclusion), week 0, week 2, week 4, and week 5. Baseline evaluation included medical history and physical examination. Venous blood samples were drawn at each study visit for routine haematological and biochemical tests and assessment of autoantibodies and complement. Fatigue was assessed at all study visits, and depression was assessed at baseline and week 5. All study visits took place at Stavanger University Hospital.

### Measures

The FSS and a fatigue visual analogue scale (VAS) were used to assess fatigue. The FSS is a generic, unidimensional fatigue instrument [23], which has been validated and is used in a number of diseases including pSS [24]. The patient evaluates 9 items, giving each item a score of 1 to 7. The FSS score is the mean score and a higher FSS score indicates more fatigue. A FSS score of 3 is commonly used as a cut-off value for fatigue in systemic lupus erythematosus (SLE) and was applied in this study. The fatigue VAS used was a horizontal 100 mm line with vertical anchoring lines. The description used at the left end (0 mm) was “No fatigue” and at the right end (100 mm) was “Fatigue as bad as it can be”.

Mood was assessed with the BDI, which is a widely used instrument to evaluate the current level of depression. A BDI score below 13 represent no depression, while a score of 14–19 reflects mild depression. A cut-off score of ≥20 was used to exclude moderately to severely depressed individuals. Mood was only assessed at inclusion and at week 5.

### Study drug and randomisation

Patients were randomly assigned to receive double-blinded therapy with anakinra (Kineret™, BioVitrum AB, SE-112 76 Stockholm, Sweden) 100 mg/day or a placebo (0.9% NaCl in identical syringes) for four weeks. Anakinra or the placebo was injected subcutaneously over 30 seconds in the abdominal or thigh region. The assigned treatment was withheld when infection or fever was present. The first injection was given at week 0 and the last at week 4. Endpoints were predetermined to be measured at week 4, as we expected fatigue to return to baseline at week 5, due to the short half-life of anakinra. Allocation of the participants was performed after complete inclusion using a computer generated randomisation list administered by the hospital pharmacy. Simple randomisation was used, with a 1∶1 allocation to either active drug or placebo. Neither the patients, investigators, nor the study nurses were aware of the assigned treatment.

The study had to be conducted in two phases due to initial problems with placebo-production; a pilot study in 2008 and the main study in 2010. It was not possible to produce the placebo in syringes identical to the active drug in 2008; therefore, all patients received their daily injections at the hospital. One of the investigators was present at every study visit during the four weeks of the study and supervised the drug injections made by one of two study nurses. The participants, nurses, and investigators were blinded to the randomisation. A research nurse, who was unblinded and not involved in patient handling, prepared the active drug and placebo in identical syringes for injection. Safety assessments were completed at every visit, and there were no missing cases. The results of the 2008 pilot study were blinded until interim analysis in 2009, revealing a non-significant trend towards a reduction in fatigue in the patients on the active drug.

The hospital pharmacy could produce placebo in syringes identical to the active drug in 2010, with a durability that allowed the patients to receive a 14 days supply of the assigned treatment at week 0 and week 2. The patients were trained to self administer the injection of either active drug or placebo, and administered the first injection under supervision at week 0. The patients registered the time of every injection, and the registration form and the empty syringes were collected at the study visit at week 2 and week 4. Safety assessments were completed at every visit. Due to infections, two patients failed to attend, one at week 4 and one at week 5.

### Outcome measures

The primary outcome measure was a group-wise comparison of fatigue scores at week 4, adjusted for baseline values. Secondary outcome measures were change in fatigue scores within each treatment group during the study, and safety and tolerability of anakinra in pSS. The proportion of patients achieving a 50% reduction in fatigue from baseline to week 4 in the active treatment group, as compared to the placebo group, was analysed as a post-hoc outcome. We regarded this as a robust measure as a 20% reduction in fatigue VAS score had been used in another recent study on rituximab-treated pSS patients [25].

### Blood analysis

Blood samples were collected at baseline, week 0, week 2, week 4, and week 5. Routine haematological tests and biochemical tests, including differential blood cell count, haemoglobin, CRP, ESR, creatinine, glomerular filtration rate (GFR), ASAT, and ALAT were performed at the hospital's analytical laboratory. Antinuclear antibodies (ANA) and antibodies to SSA/Ro and SSB/La were analysed as previously described [26].

### Sample size calculation

Based on a prior study of anakinra treatment for fatigue in RA [20], we estimated that 30 patients were needed to detect a difference in 25 points on the fatigue VAS with a power of 80% and a two-sided α of 0.05.

### Statistics

The results from the 26 patients were analysed as one cohort. Results are reported as mean ± standard deviation (SD) when normally distributed, otherwise as median and range. The t-test and Fisher's exact test were used for the comparison of continuous and categorical data, respectively. ANCOVA was used to compare fatigue scores at week 4 between groups. Due to the small sample size and the distribution of data, the non-parametric Friedman Test for repeated measures was used to analyse changes over time within groups. A p-value of <0.05 was defined as statistical significant. All analyses were carried out using SPSS for Windows version 16.0.

## Results

**Figure 2:**
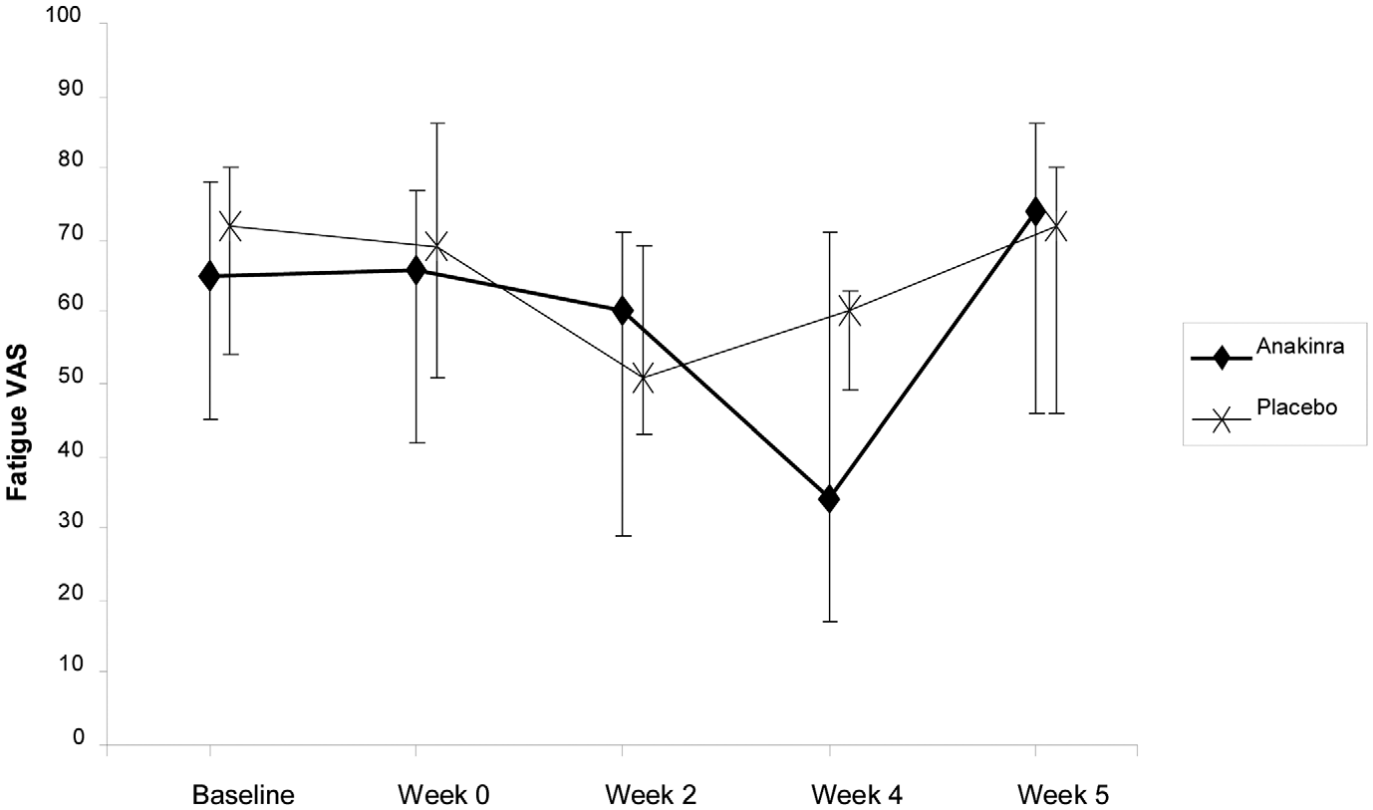
Median fatigue at baseline and during the study. Brackets represent inter-quartile range. VAS, visual analogue scale.

Thirteen of the 26 patients included were randomised to receive the active drug and 13 the placebo. Baseline characteristics for the two groups are reported in Table 1. One patient in the active drug group did not show up at week 4; thus, 12 patients in this group and 13 patients in the placebo group were available for the per-protocol analysis. There was no significant difference between the groups in fatigue score at week 4 when adjusting for baseline values, p = 0.19 (ANCOVA). There was a highly significant improvement in fatigue at week 4, compared to baseline, in the group on the active drug (p = 0.005), and an almost significant improvement in the placebo treated group, p = 0.053 (Friedman Test), Figure 2. The mean improvement in fatigue VAS at week 4 was 37% (SE 10.2%) in the active drug group and 13.5% (SE 8.0%) in the placebo group. Six out of 12 patients (50%) on the active drug and one out of 13 patients on placebo reported >50% reduction in fatigue VAS score from baseline to week 4, p = 0.03 (Fisher's exact test). The relative risk of experiencing a 50% reduction in fatigue was 6.5 (95% CI 0.9 to 46.4) for the patients in the active drug group compared to the placebo group.

**Table 1:** Selected demographic and laboratory variables at inclusion for 26 patients with primary Sjögren's syndrome (pSS).

	Active drug	Placebo
No. of subjects	13	13
Gender (Male/Female)	4/9	3/10
Age at baseline, years*	55 [36–80]	54 [32–75]
Disease duration, years*	5 [1–17]	8 [0–16]
BMI, kg/m^2^ ** †	23.2±3.4	22.5±2.4
Haemoglobin, g/L**	139±14	137±12
Leukocytes 10^9^/L**	5.5±1.5	6.2±1.3
CRP mg/L**	2.1±2.3	3.1±3.4
ESR mm/h**	10±6	11±7
TSH mIU/L**	2.1±0.8	1.8±0.9
Anti-SSA/Ro antibody (%)	13 (100)	10 (77)
Anti-SSB/La antibody (%)	7 (54)	6 (46)
Disease modifying treatment	8 (62)	9 (69)
Antimalarial drugs (%)	4 (31)	7 (54)
Prednisolone (%)	5 (38)	2 (15)
Azathioprine (%)	4 (31)	1 (8)
Leflunomid (%)	0 (0)	1 (8)
Thyroxin (%)	1 (8)	4 (31)
Betablockers (%)	1 (8)	2 (15)

*Represents median and range.

**Represents mean ± standard deviation (SD).

† BMI, body mass index. Available for the 18 patients in 2010 study only.

Numbers in parentheses represent percentages.

CRP, C-reactive protein; ESR, erythrocyte sedimentation rate; TSH, thyroid-stimulating hormone.

The intention to treat-principle specifies that all randomized patients must be included in the analysis, and according to this the fatigue VAS score at week 4 was carried forwards from the score obtained at week 2 for the patient missing at week 4. The results remained unchanged by this approach, with no difference between groups in the primary endpoint (ANCOVA, p = 0.163). The patient had a >50% reduction in fatigue VAS score and the difference in numbers of patients in each group who achieved >50% reduction in fatigue remained significant (Fisher's excact test, p = 0.03).

There were no significant changes in FSS scores in either treatment group during the study, Table 2. Fatigue levels returned to baseline one week after the last injection in both groups (week 5).

**Table 2:** Fatigue and depression scores at inclusion and during the study.

		Baseline	Week 0	Week 2	Week 4	Week 5
Fatigue VAS*	A:	65 [45–78]	66 [42–76]	60 [29–71]	34 [17–71]	74 [46–82]
	P:	72 [55–80]	69 [52–86]	51 [44–70]	60 [50–63]	72 [47–80]
FSS**	A:	5.6±1.0	5.6±1.2	5.3±1.1	5.1±1.1	5.6±0.9
	P:	5.7±0.8	5.6±1.1	4.9±1.2	4.7±1.3	5.2±1.0
BDI*	A:	5 [3–11]				4 [0–10]
	P:	8 [6–12]				7 [2–14]

*Represents median and interquartile range.

**Represents mean ± SD.

A, active drug; BDI, Beck Depression Inventory; FSS, Fatigue Severity Scale; P, placebo; VAS, visual analogue scale.

The mean change in the neutrophil cell count from week 0 to week 4 was −19.6% (SE 9.7%) in the active drug group and −12.8% (SE 5.2%) in the placebo group. However, the mean neutrophil count was still within the normal range in both groups at week 4. Complement C3 and C4 levels had a mean change of −5.4% (SE 3.0%) and −16.3% (SE 5.3%) in the active drug group, and a mean change of −2.1% (SE 1.1%) and −5.2% (SE 2.2%) in the placebo group from week 0 to week 4. Other laboratory measures, including autoantibodies, remained unchanged in both groups.

### Safety

Two serious adverse events (SAE) were observed in each group. One patient on anakinra developed a severe injection site reaction and stopped medication on day 8. She was hospitalised on day 11 due to fever, malaise, and persistent skin changes. The condition improved after treatment with antihistamines, and she could restart the medication on day 15. She was not treated with corticosteroids. Another patient in the active drug group did not attend the week 4 visit due to gastroenteritis. She improved spontaneously and no further action was taken. In the placebo group, one patient developed chest pain and was hospitalized on day 18. No cause was found and she was able to restart the medication on day 22. Another patient in the placebo group got diarrhoea on day 34 and was hospitalized. He was diagnosed with diverticulitis and treated with antibiotics.

Three patients, two on the active drug and one on placebo, had a transient episode of neutropenia. White blood cell count normalised spontaneously during continued treatment according to the protocol. Injection site reactions were common and occurred in seven of the 13 patients on the active drug (54%), and in two on placebo (15%). Apart from the one reported as a SAE, injection site reactions were transient and mild, with a burning sensation as the main symptom in the placebo group.

## Discussion

This randomised, double-blind, placebo-controlled trial of IL-1 blockade did not show a significant reduction in fatigue after treatment with IL-1 inhibition based on the primary endpoint analysis, while the post-hoc analysis indicate a possible positive effect. There are several possible explanations for this finding: One explanation is that IL-1 does not substantially influence fatigue in pSS, and that other factors leading to fatigue are more important in this setting such as depression, sleep-disorders and autonomic dysfunction [9]. As our intention was to investigate somatic factors associated with fatigue, patients with moderate to serious depression were excluded, and only one of the patients used prescription drugs against insomnia. Autonomic dysfunction was not evaluated systematically, but none of the patients spontaneously reported these types of symptoms. Therefore, we believe these factors are less important in this study.

Another explanation is that the small number of subjects led to low statistical power and therefore we could not confirm an actual effect of IL-1 inhibition on fatigue. In the post-hoc analysis, half of the patients in the active drug group did report a 50% reduction in fatigue VAS score, compared to one patient in the placebo group. Although not supported by the primary outcome measure, this may indicate that IL-1 inhibition influences fatigue in patients with pSS. This observation is in line with our primary hypothesis that a blockade of the IL-1 receptor should lead to reduction of fatigue due to reduced IL-1β signalling in the brain. This is analogous to the concept of sickness behaviour in animals, which is predominantly incited by IL-1β. The intratechal production of IL-1β is thought to reflect the peripheral production but at a significantly lower concentration [27]. IL-1β induces inflammation in the periphery, while IL-1β inside the BBB acts as a transmitter substance which causes behavioural effects without major brain inflammation [28].

We previously documented an increased concentration of the naturally occurring IL-1Ra in the cerebrospinal fluid (CSF) of patients with pSS and fatigue [7]. In cases of chronic inflammation this increased level is thought to counterbalance increased IL-1β; thus, it acts as a surrogate marker of IL-1β, which is extremely difficult to measure in CSF. Peripherally produced IL-1β can cross the BBB in selected areas by active transport and can activate the vagus and other afferent nerves with consequent signalling to the brain [27]. Further, peripherally produced cytokines activate the endothelium in the BBB, allowing neutrophiles to pass, and this may also be a major pathway for brain signaling [29]. A recent study reports how infusion of a monoclonal antibody to TNF-α inhibits pain responses in the CNS within 24 hours [30]. This monoclonal antibody does not cross the BBB, and the observed CNS effect is probably due to reduced activation of the endothelium in the BBB [30]. This indicates that a reduction in circulating levels of IL-1 in the periphery may influence fatigue signalling in the brain. In the current study, IL-1Ra was administered subcutaneously, but it also has the potential to cross the BBB by a saturable transport system [16], [31], [32].

The pSS patients included in this study showed large variations in fatigue scores at inclusion and during the study, and we believe this is the main reason we were not able to show a significant reduction in fatigue in the primary outcome measure. Also, according to the sample size calculation, 30 patients were needed to show a 25 point improvement on fatigue VAS, but for practical reasons this many patients could not be included.

FSS scores were constant in both treatment groups during the study, a phenomenon possibly explained by descriptive scales being less sensitive to change than visual analogue scales [33]. Another explanation for the lack of change in FSS is that it measures a dimension of fatigue not influenced by IL-1 modulation.

There is no established treatment regimen for fatigue in autoimmune diseases. Dass and colleagues recently investigated the effect of the B-cell depleting agent, rituximab, on fatigue in 17 pSS patients. This study found that seven out of eight patients treated with rituximab and five out of nine patients treated with placebo reported a >20% reduction in fatigue VAS at six months [25]. Another study of rituximab in pSS also reported an improvement in fatigue as measured by the Multidimensional Fatigue Inventory (MFI) [34]. Similar findings are reported in other autoimmune diseases, and the TNF-α inhibitor etanercept was shown to relieve fatigue in a cohort of psoriasis patients [35]. These findings, together with the current results, indicate that one mechanism for fatigue in pSS is immune activation. The use of biological drugs is therefore an exciting new approach to fatigue treatment in chronic inflammatory diseases. This class of drugs may influence fatigue generating pathways.

Several studies point to an influence of IL-1 on mood and depression [36]. Depressed patients were excluded from the current study because we wanted to explore the influence of IL-1 on fatigue in non-depressed patients. However, we believe it would be of great interest to map the effects of newer biological drugs on depression. Future studies of biological drugs in pSS should ideally include measures of both fatigue and depression.

### Safety

Based on our limited study results, anakinra appears to be safe in pSS patients. Side effects or SAEs in the active drug group were not more prevalent than in the placebo group. However, as the study included only 13 patients in the active drug group, and treatment was given for four weeks only, it is not possible to estimate the long-term safety of anakinra in pSS.

### Strengths and limitations

There are several limitations to this study. The number of subjects is small; reflecting the difficulty in selecting patients not biased by depression, drugs, or other factors that could influence fatigue [24]. The patients included may not represent the whole pSS cohort, as the percentage of male patients (27%) was higher than expected, and most patients were using at least one disease modifying drug. Also, we used a simple randomisation, which may have lead to unbalanced arms. In a small study like this it would at best be possible to stratify the subjects on one, possibly two variables [37]. However, it is not clear which variables are the optimal ones to employ. There is no known association in pSS between fatigue and age, disease duration, laboratory values or other clinical parameters that could be used for stratification at inclusion. Neither did we stratify according to fatigue score, as FSS>3 was a criteria for inclusion. For this reason, we decided a 1∶1 allocation was the best approach.

We did not investigate the relationship between social factors and fatigue, as the small patient number did not allow subgroup analysis. We used two instruments to measure fatigue; the fatigue VAS and the FSS. Both of these scales are unidimensional, and the use of a multidimensional scale might have provided extra information on the origin of fatigue.

The placebo effect was quite strong in this study, as both groups had a reduction in fatigue at week 2 close to significance. This is not unexpected. Figure 2 illustrates how the placebo group reported more fatigue at week 4, while the treatment group had a further decline in fatigue. We interpret this as a reduction in the placebo-effect at week 4.

We did not measure pSS disease activity during the study. The lack of a disease activity instrument that is sensitive to change has been a limitation in intervention studies of pSS patients. Recently an instrument for this purpose was developed and it is reportedly accurate in detecting changes in disease activity [38].

Local skin reactions are common following anakinra injections and are well known to patients. Thus, some patients may have guessed their allocated treatment; we can not exclude the possibility that this may have affected the final results. However, two of the patients in the placebo group also reported skin reactions.

This study was conducted in two phases. Theoretically, there may have been different patient handling and different results in the two phases. Four patients were randomised to active drug and four to placebo in 2008; in 2010, there were nine patients in each group. If only the results from the 2010 study are analysed, four out of eight patients receiving anakinra versus one out of nine patients receiving the placebo demonstrated a ≥50% reduction in fatigue from baseline, with a trend towards statistical significance when comparing the groups (p = 0.09).

Strengths of the study are the study design, the inclusion of only well characterised patients according to the 2002 American-European Consensus Group criteria and that all patients completed the assigned treatments with none lost to follow-up. There is a general lack of treatment-studies in pSS, and a lack of studies of fatigue-specific treatment in particular. This study brings new knowledge to the field. The study is fully investigator initiated and represents a novel approach to fatigue research.

## Supporting Information

Protocol S1 **Trial Protocol.**
(DOC)

Checklist S1 **CONSORT Checklist.**
(DOC)
