# Supplementary material for: Interleukin-1 Inhibition and Fatigue in Primary Sjögren's Syndrome – A Double Blind, Randomised Clinical Trial
Source: PLoS One. 2012 Jan 10;7(1):e30123. doi: 10.1371/journal.pone.0030123 (PMC3254637; doi:10.1371/journal.pone.0030123)
Supplement: Protocol S1 — Trial Protocol. (DOC) [file pone.0030123.s001.doc]

# FORSKNINGSPROTOKOLL

En dobbeltblind, placebokontrollert, parallellgruppe studie av interleukin-1 blokade som behandling av fatigue ved primært Sjøgrens syndrom – en pilotstudie

# Sammendrag

Fatigue (sykelig trøtthet) er et uttalt og invalidiserende fenomen hos mange pasienter med primært Sjøgrens syndrom (PSS). Interleukin-1 (IL-1) synes viktig for utvikling av sykdomsadferd og fatigue. Anakinra er en IL-1 reseptorantagonist som i innledende studier på pasienter med leddgikt har vist positiv effekt på fatigue. I en dobbelt-blindet studie skal anakinra eller fysiologisk saltvann gis i en måned til 30 pasienter med PSS. Effekten på fatigue evalueres ved standardiserte spørreskjema og intervju.

**Prosjektleder**

Professor Roald Omdal, dr med, overlege, Seksjon for klinisk immunologi, Medisinsk klinikk, Stavanger Universitetssjukehus

**Prosjektmedarbeidere**

Katrine Brække Norheim, cand med, ass lege, Medisinsk klinikk, Stavanger Universitetssjukehus, Erna Harboe, cand med, ass lege, Medisinsk klinikk, Stavanger Universitetssjukehus Lasse Gøransson, PhD, overlege, Medisinsk klinikk, Stavanger Universitetssjukehus Jan Terje kvaløy, statistiker, UiS, Professor Anne Marit Mengshoel, dr.philos, Seksjon for Helsefag, Medisinsk Fakultet, Universitetet i Oslo

# Introduksjon

PSS er en kronisk autoimmun sykdom der eksokrine kjertler slik som tårekjertler og spyttkjertler utgjør et vesentlig mål for det immunologiske angrepet. Det kliniske bildet domineres av tørrhetsfenomener i øyne (keratokonjunktivits sicca) og munn (xerostomi), og av uspesifikke symptomer slik som muskel- og leddsmerter (1). Det er i dag ingen effektiv behandling mot PSS, annet enn symptomatisk terapi med kunstig tårevæske, antiflogistika, og lettere smertestillende medisiner.

Fatigue (sykelig tretthet) er et mer eller mindre invalidiserende fenomen hos mange pasienter med PSS i likhet med andre kroniske inflammatoriske sykdommer (4,5). *Fatigue* kan defineres som en tilstand preget av overveldende trøtthet, mangel på energi, og følelse av utmattethet. Dette rammer også mange pasienter med multiple sklerose og kreftsykdommer (6-8). Det har blitt utført en rekke studier ved slike tilstander som viser at fatigue ofte kan være et nærmest invalidiserende fenomen. Ved SLE viser studier at omlag 80% av pasientene plages av fatigue i en slik grad at det går ut over dagliglivets aktiviteter (9,10). Selv om det finnes få sammenlignende studier, kan det se ut som fatigue ved PSS og SLE forekommer omtrent like hyppig og i samme grad. Det er videre en erfaring at fatigue ofte er årsaken til at pasienter ikke kommer tilbake til arbeidslivet, og også at behandling av grunnsykdommen ikke fører til bedring av fatigue i vesentlig grad.

Fatigue er klart assosiert til faktorer som dårlig søvn, depresjon, og andre psykososiale faktorer (5,7,10,12,13). Likevel er det påfallende hvor ofte fatigue forekommer ved kroniske inflammatoriske sykdommer. En direkte sammenheng mellom fatigue og ”faktorer assosiert med inflammasjon” er derfor en mulighet ved slike sykdommer. Hypotesen styrkes av at observasjoner hos dyr der intrathecale injeksjoner av interleukin-1β (IL-1β) medfører uttalt sykdomsadferd (14). Hos mennesker fører intravenøs injeksjon av IL-1β til fatigue, hypotensjon og uvelhet (15). Reseptorer for IL-1 (IL-1R) kan påvises i alle avsnitt av dyrehjernen (16), og hos mennesket sees oppregulering av IL-1 i hjernen ved en rekke akutte og kroniske sykdomstilstander (17). ”IL-1R familien” og dens ligander IL-1α, og IL-1β samt den naturlige forekommende reseptorantagonisten IL-1Ra synes derfor å ha viktige funksjoner både i immunsystemet og i sentralnervesystemet.

Ved PSS har cytokinprofilene blitt studert både i spytt og i serum. Både Th1- og Th2-type cytokiner uttrykkes av infiltrerende lymfocytter, og epiteliale celler produserer pro-inflammatoriske cytokiner slik som IL-1, IL-6 og TNF-α (19). Ved SLE foreligger meget få studier av cytokiner i cerebrospinalvæske, men gjennomgående synes det å foreligge økte nivåer av IL-1 og IL-6 hos pasienter med affeksjon av hjernen (20). Lignende undersøkelser foreligger ikke ved PSS, men på bakgrunn av den betydelige likhet mellom disse to tilstandene, er det naturlig å tenke seg at lignende forhold som ved SLE også er til stede ved PSS.

**Problemstilling og målsetninger**

Det er derfor holdepunkter for å anta at IL-1 spiller en direkte eller indirekte rolle for fatigue og sykdomsadferd ved sykdommer som PSS og SLE. For å teste dette, utførte vi nylig en pilotstudie på pasienter med rheumatoid artritt. Pasienter fikk IL-1R antagonist (IL-1Ra) (anakinra - Kineret®) i vanlig dosering og fatigue ble målt over en 8-ukers periode. Det kom til en kraftig og statistisk signifikant reduksjon av fatigue allerede etter 4 uker (21). Behandling med anakinra brukes vanligvis ved rheumatoid artritt, men studier har også blitt gjort på pasienter med SLE, der ingen uheldige effekter har blitt observert (22,23). Ved PSS foreligger bare mindre studier eller case-reports (Roland Jonsson – personlig meddelelse), men også her uten observerte bivirkninger eller komplikasjoner. På bakgrunn av at det ikke finnes effektiv behandling for fatigue, er det viktig å finne ut om anakinra kan ha en gunstig effekt på fatigue hos pasienter med kronisk inflammatorisk sykdom. Vi har derfor satt opp den foreliggende studien i et dobbelt-blindet, placebokontrollert parallell-gruppe design for å undersøke denne problemstillingen.

**Primære målsetninger:**

Evaluering av forskjell i gradering av fatigue innen og mellom behandlingsgruppene: Fatigue Severity Scale (FSS) (8) og en visuell analog skala for fatigue før oppstart og ved avslutning av behandlingsperioden.

**Sekundære målsetninger:**

Evaluering av forskjell i gradering av depresjon innen og mellom behandlingsgruppene: Becks Depression Inventory (28) før oppstart og ved avslutning av behandlingsperioden.

Evaluering av laboratorieprøver: Differensialtelling av blodlegemer før og hver andre uke i studien, samt bestemmelse av ASAT, ALAT, CRP, ESR, og komplementfaktorene C3 and C4. Screening for antinukleære antistoff (ANA) før og etter studien (ELISA-test).

Evaluering av IL-1 og andre relevante cytokiner (IL-2, IL-4, IL-6, IL-10) ved analyse med Luminex teknologi i vårt forskningslaboratorium.

**Statistiske metoder**

For vurdering av forskjeller mellom to grupper av kvantitative eller kvalitative variabler benyttes hhv uparret t-test (Mann Whitney ved ikke-normalfordeling) eller X^2^-test. For vurderinger av endringer av kvantitative variabler over tid benyttes Repeated Measures ANOVA.

# Forsøksobjekter og kriterier for deltakelse

Vi vil forsøke å rekruttere 30 deltakere med PSS innen Rogaland fylke. Pasientene utvelges fortløpende etter alfabetisk rekkefølge basert på etternavnet fra seksjonens polikliniske pasienter. I praksis vil dette være PSS-pasienter bosatt i Stavanger, Sandnes eller Randaberg kommune. Dette geografisk avgrensede utvalget utgjør for tiden ca 100 personer.

**Inklusjonskriterier:**

De reviderte Europeiske-Amerikanske kriterier for PSS (2), alder: 18 – 80 år, informert skriftlig samtykke.

**Eksklusjonskriterier:**

Samtidig annen sykdom som kan påvirke fatigue (ubehandlet hypo- eller hyperthyreose, malign sykdom, Parkinsons sykdom, multiple sklerose, eller annen tilstand eller sykdom der anakinra er kontraindisert, mental depresjon (skår >13 på Beck Depression Inventory) (24), FSS skår <3, anemi (Hb < 10 g/dl), nøytropeni ( <1,5 × 10^9^/liter), sykehistorie med hyppige infeksjonssykdommer, kvinner som er gravide eller ammer. Ikke adekvat prevensjon hos kvinner i fertil alder, eller ikke utført graviditetstest før oppstart av behandling.

Behandling med andre biologiske medikamenter.

**Plan for gjennomføringen**

Pasientene møter til alle kontroller på Stavanger Helseforskning AS, som ligger på sykehusets område.

**Varighet av studien:**

- Rekrutteringfase: 4 uker for kontroll av biokjemiske prøver, ledsagende sykdommer og medikamentell status.
- Behandlingsfase: 4 uker fra dag for randomisering til studiemedikament.
- Oppfølgingsfase: En etterkontroll ca 1 uke etter avsluttet behandling med studiemedikamentet.

**Studiemedikament:** Deltakerne får 100 mg (0,7 ml) anakinra eller tilsvarende volum fysiologisk saltvann (placebo) hver dag i 4 uker subkutant i tillegg til sin eventuelle ordinære medikasjon for PSS. Dosevalg baseres på effekt og sikkerhetsstudier ved bruk av 100 mg anakinra daglig hos pasienter med rheumatoid artritt. Det er ikke sannsynlig å anta at forholdene ved PSS vil være vesentlig forskjellig.

**Ledsagende medikamenter:** En stabil og lav dose kortikosteroid kan aksepteres såfremt dosen ikke planlegges justert underveis i studien.

Annen medikasjon som kan påvirke fatigue (betablokkere, anti-depressiva, eller annet) må være etablert i stabil dose senest 4 uker før studiestart og kan ikke avsluttes eller endres i løpet av studien.

**Blinding:** Aktivt medikament blir kjøpt via apoteket. Tilsvarende mengde placebo (fysiologisk saltvann) blir produsert i identiske sprøyter av Fresenius Kabi AS i Halden. Sjukehusapoteket i Stavanger re-etiketterer sprøytene i samsvar med randomiseringslisten for studien og pakker i forpakninger a 30 sprøyter per deltakernummer.

**Randomisering:** Deltakerne tildeles behandlingsgruppe fortløpende etter en forhåndsbestemt liste som fordeler deltakerne i forholdet 1:1 på anakinra eller placebo.

**Studie-prosedyrer**

Ved første kontroll i studien (rekruttering) og ved oppfølgingskontrollen etter avslutning skal det tas følgende blodprøver:

- Hgb, differentialtelling av blodlegemer, ASAT, ALAT, CRP, ESR

Umiddelbart før studiemedikamentet startes, og på dag 14 og 28 skal det tas følgende blodprøver:

- Vanlig biokjemi: Hgb, differensialtelling, ASAT, ALAT, CRP, ESR, samt immunologiske prøver: Komplementfaktorene C3 and C4, evaluering av IL-1 og andre relevante cytokiner (IL-2, IL-4, IL-6, IL-10)

Ved første kontroll i studien, 2 uker etter start av behandling, ved avslutning av studiemedikamentet og ved etterkontroll skal deltakerne fylle ut 2 spørreskjema:

- Fatigue Severity Scale (FSS), samt en visuell analog skala for fatigue.

Becks Depression Inventory fylles ut ved screening og ved avslutning av medikasjonen. Graviditetstest vil bli utført hos kvinner i fertil alder før inklusjon i studien.

**Dataregistrering:** Data nedtegnes fortløpende på papirdokumenter, og vil senere overføres til database.

**Diskontinuering av studiemedikamentet:** Deltakere som utvikler nøytropeni (<1,5 × 10^9^/liter) vil bli tatt av studiemedikamentet. For deltakere som diskontinuerer studiemedikamentet vil det samtidig bli gjort en datainnsamling som ved fullført observasjonsfase. Deltakeren vil deretter møte til en oppfølgingskontroll etter 1 uke. Dersom en deltaker må tas av studiebehandling før observasjonsfasen er fullført, vil vedkommende bli erstattet med en ny deltaker i samme behandlingsgruppe.

**Uønskede hendelser:** Alle uønskede hendelser registreres ved kontrollundersøkelsene på Stavanger helseforskning eller per telefon, og meldes samlet i sluttrapport til SLK.

**Statistisk grunnlag for studien**

I vår tidligere pilotstudie (21) på pasienter med RA fant vi en mean fatigue verdi på 74 (SD 22) bedømt ved visuell analog skala. Etter 4 ukers behandling falt denne til 38 (SD 25), dvs en forskjell på 36 mm. Dersom vi antar at endel av effekten i den aktuelle studien skyldtes ”placebo-effekt” av daglige injeksjoner vil vi forvente en mindre forskjell mellom behandlet gruppe og ubehandlet gruppe. Med utgangspunkt i dette tenker vi oss en forventet forskjell på 25 mellom behandlet og ubehandlet gruppe etter 4 uker. Vi trenger da 10 pasienter i behandlingsgruppen og 20 pasienter i placebogruppen ved en 2 sidig α 0.05 og beregnet power på 80% for studien. Dersom vi ikke forventer noen vesentlig placeboeffekt vil vi trenge et betraktelig mindre antall pasienter (6 pasienter i hver gruppe dvs til sammen 12, ved valgt 2-sidig α 0.05 og en beregnet power på 80%).

**Etiske betraktninger**

PSS er ikke en godkjent indikasjon for Anakinra. Dersom pasienter responderer godt på slik behandling, kan dette bli et etisk problem ved at det ikke foreligger refusjon ved ønske om fortsatt behandling utover behandlingsperioden. Pasientene vil derfor bli informert om at preparatet ikke kan gis med refusjon etter studieperioden. Halvparten av deltakerne vil med sikkerhet ikke oppnå medikamentell effekt. Imidlertid finnes det ikke noe behandlingsalternativ for å redusere fatigue ved PSS og deltakerne som mottar placebo vil derfor ikke få et forringet behandlingstilbud. Et eventuelt positivt utfall av denne pilotstudien vil derfor være av vesentlig verdi for den videre strategien for å utvikle tiltak for denne pasientgruppen. På denne bakgrunnen vurderes studien som etisk forsvarlig å gjennomføre.

**Relevant litteratur**

1. Jonsson R, Bowman S, Gordon TP: Sjögren’s syndrome. In: Arthritis and Allied Conditions – A Textbook of Rheumatology. Koopman WJ, Moreland LW (eds.), Lippincott, Williams & Wilkins, Philadelphia, (2005):1681-1705.

4. Wolfe F, Hawley DJ, Wilson K. The prevalence and meaning of fatigue in rheumatic diseases. J Rheumatol 1996;23:1407-17.

5. Krupp LB, LaRocca NG, Muir J, Steinberg AD. A study of fatigue in systemic lupus erythematosus. J Rheumatol 1990;17:1450-2.

6. Roelcke U, Kappos L, Lechner-Scott J, et al. Reduced glucose metabolism in the frontal cortex and basal ganglia of multiple sclerosis patients with fatigue. Neurology 1997; 48: 1566-1571.

7. Krupp LB, LaRocca NG, Muir-Nash J, Steinberg AD. The fatigue severity scale. Application to patients with multiple sclerosis and systemic lupus erythematosus. Arch Neurol 1989;46:1121-3.

8. Smets EM, Garssen B, Schuster-Uitterhoeve AL, de Haes JC. Fatigue in cancer patients. Br J Cancer 1993;68:220-4.

9. Omdal R, Mellgren SI, Koldingsnes W, Jacobsen EA, Husby G. Fatigue in patients with systemic lupus erythamtosus: lack of associations to serum cytokines, antiphospholipid antibodies, or other disease characteristics. J Rheumatol 2002;29:482-6.

10. Tench CM, McCurdie I, White PD, D`Cruz DP. The prevalence of fatigue in systemic lupus erythematosus. Rheumatology 2000;39:1249-54.

11. Godaert GL, Hartkamp A, Geenen R, Garssen A, Kruize AA, Bijlsma JW, Derksen RH. Fatigue in daily life in patients with primary Sjogren's syndrome and systemic lupus erythematosus. Ann N Y Acad Sci. 2002 Jun;966:320-6.

12. Wang B, Gladman DD, Urowitz MB. Fatigue in lupus is not correlated with disease activity. J Rheumatol 1998;25:892-5.

13. Bruce IN, Mak VC, Hallett DC, Gladman DD, Urowitz MB. Factors associated with fatigue in patients with systemic lupus erythematosus. Ann Rheum 1999;58:379-81.

14. Kelley KW, Hutchison K, French R, et al. Central interleukin-1 receptors as mediators of sickness. In: Moore PM, Lahita RG (eds.): Neuropsychiatric manifestations of systemic lupus erythematosus. Annals of the New York Academy of Sciences 1997; 823:234-46.

15. Rinehart J, Hersh E, Issell B, Triozzi P, Buhles W, Neidhart J. Phase 1 trial of recombinant human interleukin-1 beta (rhIL-1 beta), carboplatin, and etoposide in patients with solid cancers: Southwest Oncology, Group Study 8940. Cancer Invest 1997;15:403-10.

16. Farrar WL, Killian PL, Ruff MR, Hill JM, Pert CB. Visualization and characterization of interleukin-1 receptors in brain. Endocrinology 1987; 139: 459-63.

17. Konsman JP, Blond D, Vigues S. Neurobiology of interleukin-1 receptors: getting the message. Eur Cytokine Netw 2000;11:699-702.

19. Mitsias DI et al. The Th1/Th2 cytokine balance changes with the progress of the immunopathological lesion of Sjøgren’s syndrome. Clin Exp Immunol 2002;128:562–8

20. Alcocer-Varela J, Aleman-Hoey D, Alarcon-Segovia D. Interleukin-1 and interleukin-6 activities are increased in the cerebrospinal fluid of patients with CNS lupus erythematosus and correlate with local late T-cell activation markers. Lupus 1992;1;111-7.

21. Omdal R, Gunnarsson R. The effect of interleukin-1 blockade on fatigue in rheumatoid arthritis--a pilot study. Rheumatol Int. 2005;25:481-4.

22. Moosig F, Zeuner R, Renk C, Schroder JO. IL-1RA in refractory systemic lupus erythematosus. Lupus. 2004;13:605-6.

23. Ostendorf B, Iking-Konert C, Kurz K, Jung G, Sander O, Schneider M. Preliminary results of safety and efficacy of the interleukin 1 receptor antagonist anakinra in patients with severe lupus arthritis. Ann Rheum Dis. 2005;64:630-3.

24. Beck AT, Steer RA. Beck Depression Inventory Manual. San Antonio: The Psychological Corporation, 1987.
